# Identifying Key Genes of Proanthocyanidin Intervention in Fluoride-Induced Liver Injury: Integrated Molecular Docking and Experimental Validation

**DOI:** 10.3390/genes16091037

**Published:** 2025-08-31

**Authors:** Zhiyu Wu, Menghuan Xiao, Zelin Gong, Benjie Wang, Wenxin Zhao, Yiyuan Guo, Lu Yang

**Affiliations:** 1 Department of Preventive Medicine, School of Medicine, Shihezi University, Shihezi 832002, China; wuzhiyu@stu.shzu.edu.cn (Z.W.);; 2 Key Laboratory for Prevention and Control of Emerging Infectious Diseases and Public Health Security, The Xinjiang Production and Construction Corps, Shihezi 832002, China

**Keywords:** proanthocyanidin, sodium fluoride, liver injury, gene expression, gene-mRNA regulatory network, molecular docking

## Abstract

**Objectives**: The objectives of this study are to investigate the therapeutic targets and mechanisms of proanthocyanidins in alleviating fluoride-induced liver injury through network pharmacology and animal experimental validation and to explore the medicinal value of grape seed proanthocyanidins. **Methods**: Potential targets of proanthocyanidins were predicted using databases such as PubChem, SwissTargetPrediction, and GeneCards, and disease-related targets of fluoride-induced liver injury were retrieved to identify common targets between proanthocyanidins and fluoride-induced liver injury. The STRING database was utilized to construct a protein–protein interaction network, and key targets were analyzed for network topology using Cytoscape software. GO and KEGG enrichment analyses were performed on core target genes to explore the potential molecular mechanisms by which proanthocyanidins alleviate fluoride-induced liver injury. The Genes-miRNA interaction network was generated using Networkanalyst, and the molecular docking results between active components and key targets were validated using the CB-Dock2 visualization tool. In the academic context, a rat model of chronic fluoride poisoning was successfully established by means of intragastric administration of sodium fluoride. The protein expression levels of p-mTOR, p-p70s6, p62, LC3-II, and PARP1 in rat liver tissues were detected via Western blot analysis. **Results**: Network pharmacological analysis successfully identified 96 key genes, through which proanthocyanidins mitigate fluoride-induced liver injury. KEGG enrichment analysis predicted that proanthocyanidins mainly exert their therapeutic effects through the mTOR signaling pathway. The molecular docking results further demonstrated strong binding affinities between proanthocyanidins and key targets, including mTOR and PARP1. The in vivo experimental results indicate that, compared with the control group, the protein expression levels of p-mTOR, p-p70s6k, and p62 in the liver tissues of rats exposed to sodium fluoride significantly increase. Conversely, the protein expression levels of LC3-II and PARP1 significantly decrease (*p* < 0.05). The outcome of liver intervention with proanthocyanidins is exactly the opposite. **Conclusions**: Proanthocyanidins can effectively alleviate fluoride-induced liver injury, potentially by regulating the mTOR signaling pathway, autophagy, and apoptosis mechanisms. This study provides valuable insights into the protective effects of proanthocyanidins against fluoride-induced hepatic damage and offers a theoretical basis for further research in this field.

## 1. Introduction

Fluorine (F) mainly occurs in nature in the form of fluorite (CaF_2_), and it shows an uneven distribution in the environment and has high solubility [1]. The intake of fluoride has a dual effect on human health: under low-concentration conditions, fluoride ions can deposit in bone tissue, thereby enhancing its structural stability; however, excessive fluoride exposure via sources such as drinking water, food, toothpaste, environmental pollutants, and others may negatively impact both skeletal and dental health [2]. Endemic fluorosis is defined as a systemic condition caused by the chronic accumulation of excessive fluoride ions in the body, leading to significant damage to bone tissue and characterized primarily by dental fluorosis and skeletal fluorosis as the main clinical manifestations [3]. Excessive fluoride intake disrupts the normal physiological functions of the liver [4]. As a key organ involved in xenobiotic metabolism, the liver is particularly vulnerable to fluoride-induced injury, which can lead to degenerative changes and impaired detoxification capacity [5]. Excessive fluoride intake causes notable morphological alterations in the liver, such as central venous congestion, inflammatory cell infiltration, hepatocyte swelling, and degeneration [6]. These pathological changes activate oxidative stress responses, resulting in reduced antioxidant enzyme activity and an elevated risk of hepatocyte damage [7].

Autophagy is a highly conserved cellular degradation mechanism in hepatocytes and plays a pivotal role in the pathogenesis and progression of liver diseases. It is evolutionarily conserved and essential for maintaining normal hepatic physiological functions [8]. Autophagy precisely degrades and recycles cytoplasmic components, which is critical for sustaining cell viability and homeostasis. Alterations in autophagy levels can disrupt the normal physiological functions of hepatocytes [9]. When cellular autophagy is impaired, a cascade of pathological events is initiated. Damaged organelles and excessive or harmful cellular components progressively accumulate. Once these reach a critical threshold, they may trigger cell death [10].

Proanthocyanidins (PCs) are a class of biologically active flavonoid compounds characterized by a distinct molecular structure, primarily composed of polymers formed from catechin and epicatechin monomers. These compounds exhibit strong antioxidant properties, effectively protecting cells from oxidative stress-induced damage [11]. Grape Seed Proanthocyanidin Extract (GSPE) is particularly abundant in proanthocyanidins, with a purity level reaching up to 95% [12]. GSPE has been demonstrated to enhance hepatocyte autophagy, reduce hepatic lipid accumulation, and suppress excessive reactive oxygen species (ROS) production, thereby alleviating fluoride-induced liver injury [13]. This study aims to utilize network pharmacology to predict potential key targets and associated molecular pathways involved in GSPE-mediated protection against fluoride-induced liver injury. Molecular docking validation and in vivo experiments using a chronic fluorosis rat model will be performed to clarify the role of GSPE in the pathogenesis and progression of fluoride-induced hepatic damage. By elucidating the underlying molecular mechanisms and developing targeted intervention strategies, this research seeks to optimize the utilization of grape-derived resources and provide a scientific basis for the prevention and treatment of related diseases.

## 2. Materials and Methods

### 2.1. Animal Rearing and Treatment

Sixty 8-week-old adult Sprague-Dawley (SD) rats weighing 180–220 g (female: male ratio = 1:1) were obtained from the Experimental Animal Center of Xinjiang Medical University (Production License No. SCXK [Xin] 2018-0002). The animals were housed in the Translational Medicine Laboratory of Shihezi University, and the experimental protocol was approved by the Medical Ethics Committee of the First Affiliated Hospital of Shihezi University (Animal Ethics Approval No. A2023-240-01). Throughout the experimental period, the animal room was maintained at 20–25 °C with 50–60% humidity under a 12 h light/dark cycle. Rats were housed in polysulfone plastic cages with ventilated wire mesh tops. After one week of acclimatization, the rats were randomly divided into six groups (n = 10 per group, equal numbers of males and females) based on body weight: control group (ad libitum access to double-distilled water with fluoride concentration < 1.5 mg/L, gavage with double-distilled water), NaF exposure group (ad libitum access to 100 mg/L fluoride water, gavage with double-distilled water), high-dose GSPE intervention group (ad libitum access to double-distilled water, gavage with 400 mg/kg·bw GSPE), and three combined intervention groups of NaF exposure with low-, medium-, and high-dose GSPE (ad libitum access to double-distilled water, gavage with 100 mg/kg·bw, 200 mg/kg·bw, and 400 mg/kg·bw GSPE, respectively). All groups were fed standard diet ad libitum, with bedding changed every three days and body weight measured weekly. (Body weight records are presented in Table S1.) After two months, rats were paired (1:1 male: female), and pregnant rats were individually housed with continued exposure until weaning at 21 days post-partum. Offspring were exposed using the same protocol as their parents until two months of age, after which they were euthanized for collection of blood and liver tissue samples, which were stored at −80 °C.

### 2.2. Main Reagents and Instruments

Main reagents: Proanthocyanidins (batch number: P823328) were purchased from Shanghai Macklin Biochemical Technology Co., Ltd. (Shanghai, China); Sodium fluoride (batch number: S111586) was purchased from Shanghai Aladdin Biochemical Technology Co., Ltd. (Shanghai, China); Alanine aminotransferase (ALT) and aspartate aminotransferase (AST) detection kits (batch number: C009) were purchased from Nanjing Jiancheng Bioengineering Institute (Nanjing, China); The phospho-mTOR (Ser2448) monoclonal antibody was purchased from the United States-based Proteintech Company (Rosemont, IL, USA) (Cat No. 67778-1-Ig, 1:10,000, 10 mL TBST); The Phospho-p70 S6 Kinase (Thr389/Thr412) Antibody was purchased from the Affinity Company (Brisbane, Australia) (Cat No. AF3228, 1:2000, 10 mL TBST); PARP1 Recombinant Rabbit Monoclonal Antibody was purchased from the HUABIO Company (Woburn, MA, USA) (Cat No. ET1608-56, 1:2000, 10 mL TBST); The P62/SQSTM1 Recombinant antibody was purchased from the United States-based Proteintech Company (Cat No. 84826-1-RR, 1:5000, 10 mL TBST); The LC3 Polyclonal antibody was purchased from the United States-based Proteintech Company (Cat No. 14600-1-AP, 1:2000, 10 mL TBST); The GAPDH Monoclonal antibody was purchased from the United States-based Proteintech Company (Cat No. 60004-1-Ig, 1:50,000, 10 mL TBST); The Multi-rAb™ HRP-Goat Anti-Mouse Recombinant Secondary Antibody (H+L) was purchased from the United States-based Proteintech Company (Cat No. RGAM001, 1:10,000, 10 mL TBST); The Multi-rAb™ HRP-Goat Anti-Rabbit Recombinant Secondary Antibody (H+L) was purchased from the United States-based Proteintech Company (Cat No. RGAR001, 1:10,000, 10 mL TBST); RIPA cell lysis buffer (batch number: R0010), Loading buffer (batch number: P1015), and protease phosphatase inhibitor mixture (PMSF) (batch number: P0100) were purchased from Beijing Solarbio Science & Technology Co., Ltd. (Beijing, China); TBS, PBS powder, acrylamide-bisacrylamide (Acr), Tris 6.8 (batch number: BL1979A), Tris 8.8 (batch number: BL515A), 10% SDS (batch number: BL517A), and ECL chemiluminescence kit (batch number: BL520A) were purchased from Hefei Baisha Biotechnology Co., Ltd. (Hefei, CHina); Bovine serum albumin (BSA) (batch number: 4240) was purchased from Biofroxx, China (Guangzhou, China). Main instruments: TGL-16G-A high-speed centrifuge (Xiangyi Laboratory Instrument Development Co., Ltd., Changsha, China); FRESCO 21 low-temperature centrifuge (Thermo Fisher Scientific, Waltham, MA, USA); Mini-PROTEAN Tetra electrophoresis apparatus (Bio-Rad, Hercules, CA, USA); Mini Trans-Blot electrotransfer apparatus (Bio-Rad, CA, USA); Trans-Blot 1703940 semi-dry transfer apparatus (Bio-Rad, CA, USA); XW-80A vortexer (Haimen Qilinbei Instrument Manufacturing Co., Ltd., Nantong, China); TS-1 horizontal rapid shaker (Haimen Qilinbei Instrument Manufacturing Co., Ltd.); TS-200B constant-temperature slow shaker (Wuxi Maritec Co., Ltd., Wuxi, China); SCIENTZ-IID cell disruptor (Ningbo Newchips Biotechnology Co., Ltd., Ningbo, China); Nanodrop ONE spectrophotometer (Thermo Fisher Scientific).

### 2.3. Determination of Serum ALT and AST

In accordance with the kit protocol, a standard curve was established, and the optical density (OD) values of each well were measured. These values were subsequently incorporated into the derived fitting equation from the standard curve to calculate the serum ALT and AST activities in Karmen units. Finally, the serum ALT and AST activity values were determined by applying the sample calculation formula.

### 2.4. Western Blot

Approximately 50 mg of liver tissue was collected from each group of mice and homogenized in an appropriate volume of RIPA lysis buffer. The tissue was then sonicated on ice at 80 W for 60 s, followed by centrifugation at 12,000 rpm for 20 min. The supernatant was collected, and protein concentration was determined using a spectrophotometer. Subsequently, 4× protein loading buffer was added, and the samples were denatured by heating at 100 °C for 10 min before storage at −20 °C. Based on the molecular weight of the target protein, appropriate separating and stacking gels were selected. Electrophoresis was performed at a constant voltage of 180 V for 45 min, followed by electrotransfer at a constant current of 400 mA for 50 min. The membrane was blocked with 5% skim milk at room temperature for 120 min and washed three times with TBST, each for 10 min. Primary antibody incubation was carried out overnight at 4 °C, followed by three TBST washes, each for 10 min. The membrane was then incubated with secondary antibody at room temperature for 120 min and washed three times with TBST, each for 10 min. Finally, ECL chemiluminescent reagent was applied, and the membrane was visualized using a fully automated chemiluminescence imaging system. Protein band intensity was quantified using ImageJ (version 1.54g) software.

### 2.5. Statistical Analysis

Statistical analysis and graph generation were performed using GraphPad Prism 9.0. Quantitative data were expressed as mean ± standard deviation ($\bar{x}$ ± s). Comparisons between two groups were conducted using Student’s *t*-test, while comparisons among multiple groups were analyzed using one-way ANOVA. A *p*-value of <0.05 was considered statistically significant.

### 2.6. Prediction of Potential Targets of PC

Based on the availability of the PC crystal structure, choosing PC for analysis can lead to the acquisition of an accurate crystal structure. Compared with GSPE, choosing PC for analysis can lead to the discovery of more potential targets. The chemical structure and SMILES number of proanthocyanidins were retrieved from the Pubchem database (https://pubchem.ncbi.nlm.nih.gov/). In the component-target databases including CTD (https://ctdbase.org/), GenesCards (https://www.genecards.org/), STITCH (https://ngdc.cncb.ac.cn/databasecommons/database/id/208, accessed on 27 August 2025), SwissTargetPrediction (http://www.swisstargetprediction.ch/, accessed on 1 July 2025), and TargetNet (http://targetnet.scbdd.com, accessed on 1 July 2025), by setting the keyword as “proanthocyanidins”, selecting the species as “Homo sapiens”, with a probability threshold > 0 and *p* < 0.05, the potential action targets of proanthocyanidins were obtained. The gene data from these databases were merged, and the duplicates were removed to form the target gene set of proanthocyanidins [14].

### 2.7. Prediction of Potential Targets for Fluoride-Induced Liver Injury

Disease targets for liver injury were obtained from the human gene database GenesCards (https://www.genecards.org/, accessed on 1 July 2025), the database DrugBank (https://go.drugbank.com/, accessed on 1 July 2025), the OMIM database (https://omim.org/, accessed on 1 July 2025), and the drug target database TTD (https://db.idrblab.net/ttd/, accessed on 1 July 2025). The keyword was restricted to “fluoride-induced liver injury”, and genes were selected with the species specified as “human”, a probability threshold > 0, and *p* < 0.05. The gene data from these databases were merged, and duplicate entries were removed to form the disease-target gene set for fluoride-induced liver injury. [15].

### 2.8. Construction of PPI Network and Screening of Key Targets

The VennDiagram package in R (version 4.2.1) was used to draw a Venn diagram of the intersection between the targets of proanthocyanidins and the targets of diseases, thereby obtaining the potential targets of proanthocyanidins in alleviating fluoride-induced liver injury. The STRING database (https://www.string-db.org/) for analyzing protein–protein interactions was utilized, with the species limited to human and a confidence score threshold of ≥0.4 set to construct the PPI network diagram of proanthocyanidin targets in fluoride-induced liver injury. The interaction results were imported into Cytoscape software (version 3.9.1), and key targets were screened based on different algorithms. The top 20 were identified as key targets [16].

### 2.9. GO and KEGG Enrichment Analysis

Using R (Version 4.2.1), the clusterProfiler package, Enrichplot package, and Org.Hs.eg.db package were invoked to conduct GO and KEGG enrichment analyses on the common targets of proanthocyanidins and fluoride-induced liver injury, respectively. The screening threshold was set at *p* < 0.05, and bar charts were plotted. These analyses revealed the potential roles of key genes in biological processes, molecular functions, and cellular components, as well as the signaling pathways in which they are involved [17].

### 2.10. Construction of the miRNA Regulatory Network for Key Targets

Utilize the integrated multi-omics NetworkAnalyst database (https://www.networkanalyst.ca, accessed on 1 July 2025). Select the top 3 hub genes ranked under the degree algorithm as key targets. Input the 3 key targets and perform conversion and annotation of gene IDs. Specify the species as Homo sapiens and choose the analysis type. Then, visualize the gene-miRNA regulatory network [18].

### 2.11. Molecular Docking

The amino acid sequences of key targets were retrieved from the UniProt database (https://www.uniprot.org), while the crystal structures were downloaded from the RCSB Protein Data Bank (PDB, https://www.rcsb.org/). The three-dimensional structure of PC (CID: 107876) was obtained from the PubChem database (https://pubchem.ncbi.nlm.nih.gov/). Molecular docking was performed using the CB-Dock2 tool (http://clab.labshare.cn/cb-dock2/, accessed on 1 July 2025). The PDB files of key targets and the proanthocyanidin ligand file were uploaded, with the binding pocket automatically predicted. Five independent docking runs were conducted for each target, and the conformation with the lowest binding energy (kcal/mol) was recorded as the optimal result [19]. (TP53 PDB ID: 1KZY, IL1B PDB ID: 1HIB, CASP3 PDB ID: 1CP3, IL6 PDB ID: 1ALU, ALB PDB ID: 1AO6, BCL2 PDB ID: 1G5M, PARP1 PDB ID: 1UK0, mTOR PDB ID: 1AUE).

## 3. Results

### 3.1. Prediction of Potential Targets for PC and Fluoride-Induced Liver Injury

The potential target genes of proanthocyanidins were obtained from the CTD, GenesCards, STITCH, SwissTargetPrediction and TargetNet databases, with 26, 116, 9, 23 and 62, respectively. After converting to unified gene names through the Uniprot database, all chemical components and targets were standardized and merged, and duplicates were removed, resulting in a total of 203 effective target genes. The potential target genes of fluoride-induced liver injury were obtained from the GenesCards, DrugBank, OMIM and TTD databases, with 1832, 6, 97 and 5, respectively. After converting to unified gene names through the Uniprot database, all chemical components and targets were standardized and merged, and duplicates were removed, resulting in a total of 1940 effective target genes (Tables S2 and S3).

### 3.2. Common Targets of Proanthocyanidins and Fluoride-Induced Liver Injury

**Figure 1:**
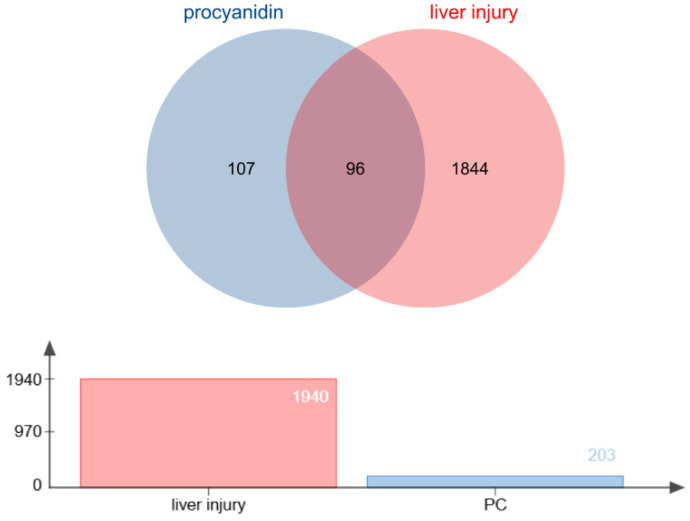
Common targets of PC and fluoride-induced liver injury.

By utilizing R and invoking the VennDiagram package, 96 common targets between proanthocyanidins and fluoride-induced liver injury were identified. These targets are considered potential core targets for the alleviation of fluoride-induced liver injury by proanthocyanidins. A Venn diagram was generated, as illustrated in Figure 1.

### 3.3. Construction of PPI Network and Screening of Key Targets

**Figure 2:**
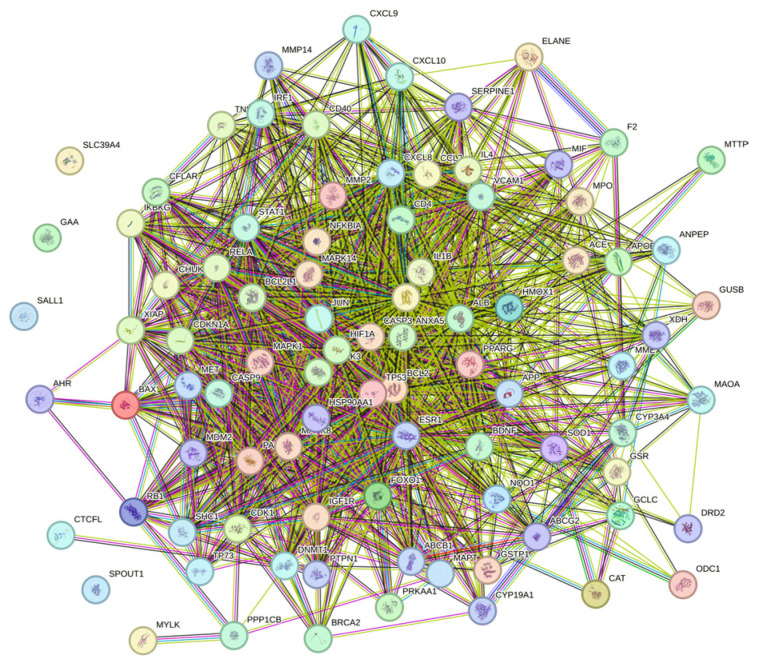
Network interaction of common targets in the STRING database.

**Figure 3:**
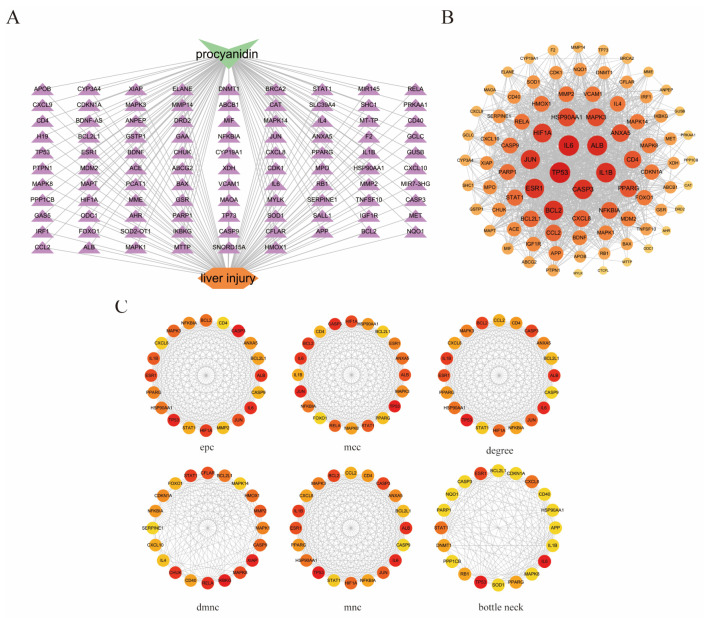
Network diagram of common targets in Cytoscape. (**A**) Network construction of common targets of proanthocyanidins and liver injury. (**B**) Core network of common targets of proanthocyanidins and liver injury. (**C**) Key targets (top 20) under six different algorithms in Cytoscape.

We will investigate the interrelationships among these 96 targets by querying the STRING database with a confidence score threshold set at ≥0.4. After filtering out isolated targets, the constructed network diagram comprises 89 nodes and 1349 edges, with an average degree score of 30.3 (Figure 2). Subsequently, the tsv file exported from STRING was imported into Cytoscape software to generate a network relationship diagram illustrating the common targets between proanthocyanidins and fluoride-induced liver injury (Figure 3A). The targets were ranked according to their degree, where darker colors and larger circles indicate stronger protein–protein interactions and greater target significance (Figure 3B). Based on the ranking of hub genes under the degree algorithm, we selected TP53, IL1B, CASP3, IL6, ALB, BCL2, and PARP1 for further analysis. Additionally, the top 20 genes ranked by importance across different algorithms are presented in the corresponding figure (Figure 3C). This visualization method provides a comprehensive overview of the interaction relationships among key targets, offering valuable insights into the molecular mechanisms underlying procyanidin-mediated alleviation of fluoride-induced liver injury.

### 3.4. GO and KEGG Pathway Enrichment Analysis

**Figure 4:**
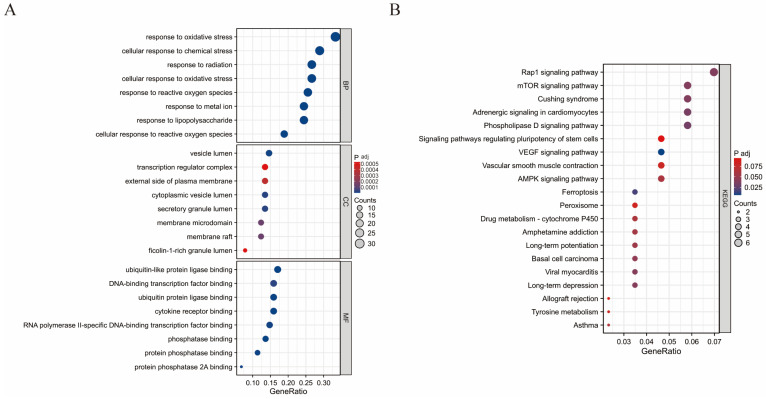
Bubble charts of GO and KEGG enrichment analysis. (**A**) Bubble chart of GO enrichment analysis. (**B**) Bubble chart of KEGG pathway enrichment analysis.

We will further investigate the interrelationships among these 96 targets. After setting the adjusted *p*-value threshold at ≤0.05, a total of 2358 entries were obtained through Gene Ontology (GO) analysis (Figure 4A), revealing that these targets are primarily enriched in three categories: Biological Process (BP), Cellular Component (CC), and Molecular Function (MF). Within BP, these genes are predominantly enriched in 2159 entries related to antioxidant response, response to reactive oxygen species, and cellular response to chemical stimuli. It is indicated that oxidative stress is the core mechanism of fluoride-induced liver injury, while PC can exert a protective effect. In CC, they are mainly concentrated in 57 entries associated with vesicle lumen, secretory granule lumen, and cytoplasmic vesicle lumen. Autophagy degrades damaged organelles and macromolecules by forming autophagic vesicles, providing cells with energy and materials for reconstruction and maintaining the stability of the internal environment, which indicates that the autophagic process may be activated. For MF, these genes are primarily enriched in 142 entries involving ubiquitin-like protein ligase binding, cytokine receptor binding, and phosphatase binding. It indicates that fluoride damage may disrupt protein homeostasis and activate harmful signaling pathways. After PC intervention, the cell’s homeostasis was restored. The top eight entries from each category were selected for bubble plot visualization. Through KEGG pathway enrichment analysis, 161 entries were identified (Figure 4B), with these targets primarily enriched in pathways such as Rap1 signaling, mTOR signaling, ferroptosis, and AMPK signaling. These findings suggest that proanthocyanidins may influence the pathogenesis and progression of fluoride-induced liver injury by modulating mechanisms related to apoptosis and ferroptosis.

### 3.5. Construction of Genes-miRNA Network for Key Targets

**Figure 5:**
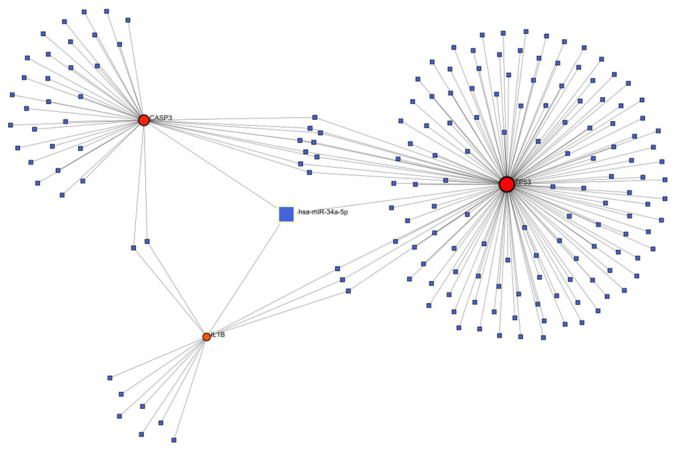
Construction of Genes-miRNA Network for Key Targets.

The Genes-miRNA interaction network was generated through NetworkAnalyst. Comprehensive experimentally verified Genes-miRNA interaction data were collected from the miRTarBase database to construct the Genes-miRNA networks of TP53, IL1B and CASP3, as shown in the figure. There are a total of 179 nodes and 192 edges. Among them, hsa-miR-34a-5p, this one miRNA, is related to the three key targets, indicating that this one miRNA can simultaneously regulate the expression of TP53, IL1B and CASP3 (Figure 5).

### 3.6. The Results of Molecular Docking

**Figure 6:**
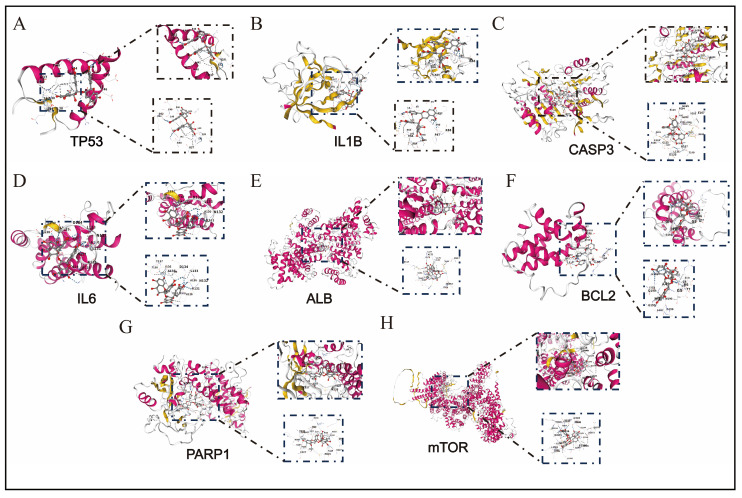
Molecular docking of proanthocyanidin ligands with 8 receptors. (**A**) Molecular docking of proanthocyanidin with TP53. (**B**) Molecular docking of proanthocyanidin with IL1B. (**C**) Molecular docking of proanthocyanidin with CASP3. (**D**) Molecular docking of proanthocyanidin with IL6. (**E**) Molecular docking of proanthocyanidin with ALB. (**F**) Molecular docking of proanthocyanidin with BCL2. (**G**) Molecular docking of proanthocyanidin with PARP1. (**H**) Molecular docking of proanthocyanidin with mTOR.

To explore the ability of proanthocyanidins to intervene in depression induced by fluoride, the targets closely related to fluoride-induced liver injury, namely TP53, IL1B, CASP3, IL6, ALB, BCL2, PARP1, and mTOR, were selected as potential targets. The predicted results of CB-Dock2 are shown in Figure 6, while the binding energies and cavity volumes of each docking group are listed in Table 1. The differences in binding energy suggest that proanthocyanidins may preferentially target PARP1 and mTOR.

### 3.7. Histological Evidence of GSPE Alleviating Liver Injury in Rats Caused by Fluoride Poisoning

**Figure 7:**
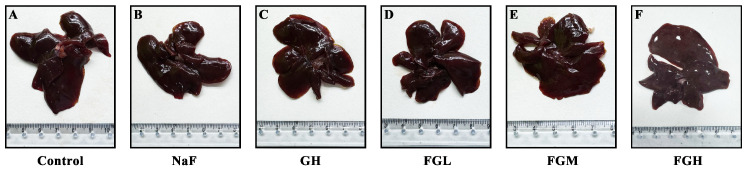
Photos of rat liver tissues in 6 groups. (**A**) Rat liver tissue in the control group. (**B**) Rat liver tissue in the NaF exposure group. (**C**) Rat liver tissue in the high-dose GSPE group. (**D**) Rat liver tissue in the NaF + low-dose GSPE group. (**E**) Rat liver tissue in the NaF + medium-dose GSPE group. (**F**) Rat liver tissue in the NaF + high-dose GSPE group.

**Figure 8:**
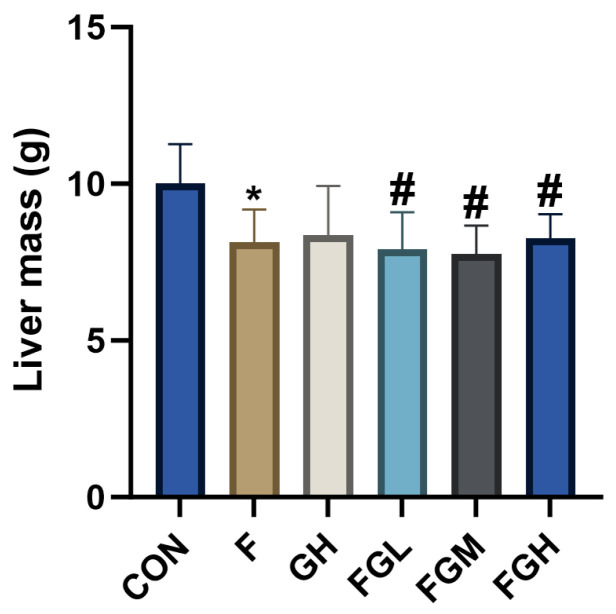
Bar charts of liver mass under different treatment groups. Compared with the control group, * *p* < 0.05. Compared with the NaF group, ^#^ *p* < 0.05.

To explore the effects of GSPE and NaF on the liver, the liver of the NaF group was observed to be dark red, with a shrunken edge and reduced volume compared to the control group, suggesting fatty degeneration or congestion. Compared to the control group, the liver volume and color of the high-dose GSPE group showed no significant difference, indicating that GSPE may not cause morphological changes in liver tissue. Compared to the NaF group, the surface color of the liver in the NaF and GSPE combined group tended to be normal reddish-brown as the GSPE dose increased, and the surface smoothness improved significantly with the increase in GSPE dose (Figure 7). As shown in Figure 8, the liver mass under fluoride exposure was lower than that of normal livers (*p* < 0.05), while the liver mass after GSPE intervention was higher than that under fluoride exposure (*p* < 0.05). This indicates that GSPE may alleviate liver damage caused by NaF exposure in a dose-dependent manner.

### 3.8. The Effects of NaF and GSPE on the Expression of ALT and AST in the Liver

**Figure 9:**
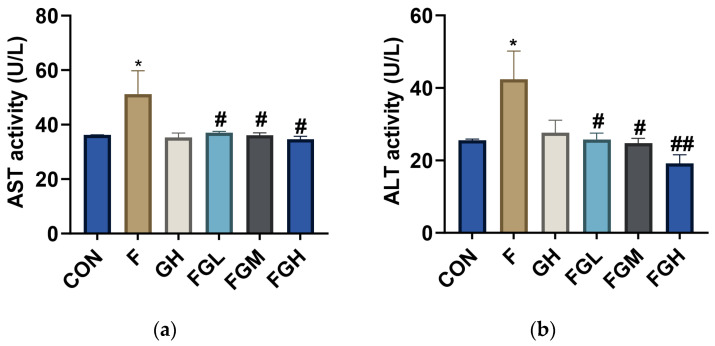
Bar charts of AST activity values and ALT activity values under different treatment groups. Compared with the control group, * *p* < 0.05. Compared with the NaF group, ^#^ *p* < 0.05, ^##^ *p* < 0.01. (**a**) AST; (**b**) ALT.

To explore the effects of GSPE and NaF on liver function, SD rats in the offspring generation were exposed to 100 mg/L NaF for 2 months, and the contents of ALT and AST in serum were determined (Table 2). Compared with the control group, the activities of AST and ALT in the NaF group were increased (*p* < 0.05). Compared with the NaF group, the activities of AST and ALT in the low-, medium-, and high-dose GSPE groups were significantly decreased (*p* < 0.05 or *p* < 0.01), and a decreasing trend was observed with the increase in GSPE intervention concentration (Figure 9). This suggests that fluoride exposure may damage liver function in rats, and GSPE may alleviate the effects of fluoride exposure on liver function.

### 3.9. The Effects of NaF and GSPE on the Expression of the mTOR/P70s6k Signaling Pathway in the Liver

**Figure 10:**
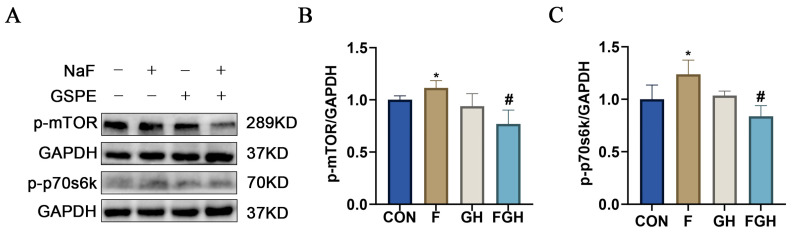
(**A**) Representative Western-blot protein bands of rat liver-related proteins (p-mTOR, p-p70s6k) under different treatment groups; (**B**,**C**) Bar charts of statistical analysis of protein gray values; compared with the control group, * *p* < 0.05, the difference was statistically significant; compared with the NaF group, ^#^ *p* < 0.05, the difference was statistically significant.

KEGG pathway enrichment analysis indicated that the mTOR signaling pathway was one of the key common targets of procyanidin and fluoride-induced liver injury, as shown by the difference in molecular docking binding energy. This protein plays a crucial role in regulating cell growth, metabolism, autophagy, and apoptosis, all of which are closely related to the pathogenesis of liver injury [20]. Given its significant enrichment and core biological significance in liver injury and protection, we hypothesized that the mTOR/p70s6k signaling pathway might be a key pathway mediating the protective effect of procyanidin against fluoride-induced hepatotoxicity. To verify this computational prediction, Western blotting was used to detect the expression of phosphorylated proteins of key components (mTOR and p70s6k) in the liver tissue of rat models. Compared with the control group, sodium fluoride treatment led to an increase in the expression levels of p-mTOR and p-p70s6k in liver tissue (*p* < 0.05). In contrast, administration of high-dose procyanidin significantly decreased the expression levels of p-mTOR and p-p70s6k in liver tissue. This indicates that GSPE may alleviate the toxic effects of sodium fluoride through the mTOR/p70s6k signaling pathway (Figure 10).

### 3.10. The Effects of NaF and GSPE on the Expression of Autophagy-Related Proteins in the Liver

**Figure 11:**
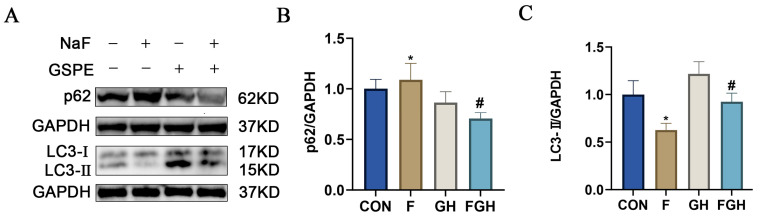
(**A**) Representative Western-blot protein bands of autophagy-related proteins (p62, LC3-II) expression in rat liver under different treatment groups; (**B**,**C**) Bar charts of statistical analysis of protein gray values; compared with the control group, * *p* < 0.05, the difference was statistically significant; compared with the NaF group, ^#^ *p* < 0.05, the difference was statistically significant.

mTOR is recognized as a major inhibitor of autophagy [21], a cellular self-degradation process that is crucial for maintaining hepatocyte homeostasis by eliminating damaged organelles and proteins [22]. We hypothesized that proanthocyanidins might alleviate fluoride-induced hepatotoxicity by restoring autophagic flux. To verify this hypothesis, we evaluated key autophagy markers: LC3-II and p62/SQSTM1, to investigate the protective effect of GSPE on liver tissues induced by NaF. Compared with the control group, the expression level of p62 in the liver tissues treated with sodium fluoride decreased (*p* < 0.05). In contrast, administration of high-dose proanthocyanidins significantly increased the expression level of p62 in liver tissues. Compared with the control group, the expression level of LC3-II in the liver tissues treated with sodium fluoride decreased (*p* < 0.05). In contrast, the use of a high dose of GSPE upregulated the expression level of LC3-II in liver tissues (Figure 11).

### 3.11. The Effects of NaF and GSPE on the Expression of Apoptosis-Related Proteins in the Liver

**Figure 12:**
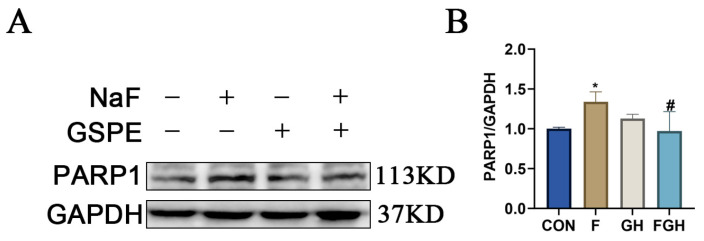
(**A**) Representative Western-blot protein bands of apoptosis-related protein (PARP1) expression in rat liver under different treatment groups; (**B**) Bar chart of statistical analysis of protein gray values; compared with the control group, * *p* < 0.05, the difference was statistically significant; compared with the NaF group, ^#^ *p* < 0.05, the difference was statistically significant.

Compared with the control group, the expression of apoptosis-related protein PARP1 in the liver tissue of the NaF group was significantly increased (*p* < 0.05), suggesting that NaF could cause apoptosis of rat liver cells. Compared with the NaF group, the expression of PARP1 protein in the liver tissue was significantly decreased after intervention with high-dose GSPE (*p* < 0.05), indicating that GSPE has a good protective effect on the liver (Figure 12).

## 4. Discussion

Fluoride is a naturally occurring inorganic anion [23]. Appropriate intake of fluoride can prevent dental caries and promote bone health [24]. However, excessive intake may induce skeletal damage such as dental fluorosis and skeletal fluorosis, and it can accumulate in various non-skeletal tissues, leading to toxic effects such as oxidative stress and cell death [25]. As the core organ for metabolism and detoxification, the liver is particularly vulnerable to fluoride toxicity [26]. The underlying mechanisms involve the accumulation of reactive oxygen species (ROS), depletion of antioxidant enzymes, and activation of processes such as apoptosis and autophagy [27].

This study systematically analyzed the potential mechanisms of proanthocyanidins in antagonizing fluoride-induced liver injury through network pharmacology approaches, identifying 10 key targets including TP53, IL1B, CASP3, IL6, and PARP1. As a crucial tumor suppressor, TP53 regulates the cell cycle, apoptosis, and metabolic reprogramming in cellular stress responses [28]. Enrichment analysis in this study indicates that it significantly participates in the apoptotic process, echoing the previously reported TP53–mTOR regulatory axis in hepatocellular carcinoma [29]. Activation of the mTOR signaling pathway suggests that TP53 may influence the balance between autophagy and apoptosis through the regulation of mTOR in fluoride-induced liver injury. Similarly, as a pro-inflammatory cytokine, IL1B can induce the expression of iNOS and the production of nitric oxide, leading to mitochondrial dysfunction, which is consistent with the mechanism of inflammation-related liver injury and further strengthens the role of the inflammatory response in fluoride-induced hepatotoxicity [30]. CASP3 encodes a cysteine-aspartic protease that plays a central role in the execution phase of apoptosis [31]. Research has revealed that the potential molecular mechanism of Confusoside in treating liver injury involves apoptosis inhibition [32], suggesting that GSPE may antagonize fluoride-induced liver injury through similar pathways.

In addition, through gene–miRNA network analysis, we found that hsa-miR-34a-5p is co-regulated by TP53, IL1B, and CASP3. Previous studies have shown that it can participate in the regulation of liver injury via the ROS/JNK/p38 pathway [33,34], suggesting that proanthocyanidins may alleviate fluorosis by influencing the expression level of this miRNA. Our molecular docking results demonstrate that proanthocyanidins exhibit favorable binding with mTOR and PARP1 (binding energies of −9.8 kcal/mol and −10.9 kcal/mol, respectively), indicating their potential to mitigate fluoride-induced liver injury through these targets.

The mammalian target of rapamycin (mTOR) serves as a crucial central regulator governing cellular energy metabolism, growth, and development [35]. It is highly sensitive to energy fluctuations. When cells are in an energy-depleted environment or experience insufficient ATP supply, they enter a starvation state [36]. In this situation, mTOR activity is inhibited, and it transmits an emergency signal to initiate autophagy. Inside the cell, the ULK1 complex begins to be recruited to form an isolation membrane. By encapsulating misfolded proteins or aged and damaged organelles within the cell, lysosomes digest them, and the hydrolyzed products are released into the cytoplasm to maintain cellular physiological functions [37]. Once the intracellular energy metabolism reaches equilibrium, mTOR activity returns to normal levels and continues to monitor changes in intracellular metabolic status. And p70s6k is one of the extensively studied substrates of mTORC1 [38], which is primarily involved in various biological processes, including mitochondrial biogenesis and mRNA transcription regulation. A study has shown that ethanol induces damage to SH-SY5Y cells by inhibiting the activity of the mTOR/p70s6k pathway. However, pretreatment with caffeine exacerbates cell damage [39], suggesting that caffeine also exerts a negative regulatory effect on the mTOR/p70s6k pathway. Our research has found that after sodium fluoride exposure in rat livers, the expression levels of p-mTOR and p-p70s6k proteins are significantly increased. After intervention with GSPE, the expression levels of these two proteins are significantly lower compared to the NaF group, indicating that GSPE has an inhibitory effect on the mTOR/p70s6k pathway.

Similarly, PARP1 is one of the key substrates of Caspase–3 [40]. Under conditions of DNA damage and energy depletion, PARP1 promotes apoptosis [41]. Fluoride can activate PARP1 by inducing mitochondrial dysfunction and oxidative stress, thereby triggering apoptosis in hepatocytes [42]. A study has found that the expression levels of both cleaved-caspase3 protein and mRNA in rat liver cells increase after long-term exposure to a fluorine environment [43]. This is consistent with the findings of our study, where sodium fluoride was found to elevate the expression of PARP1 in rat livers, indicating that sodium fluoride is hepatotoxic. After intervention with GSPE, the expression of PARP1 decreased significantly, providing strong evidence for the hepatoprotective effect of GSPE.

This study integrated key targets and pathways from the perspective of network pharmacology and connected with previous findings in other toxicity models, providing a new perspective for understanding the liver-protecting mechanism of proanthocyanidins. In the future, more experiments are needed to further verify the specific action mechanisms of these predicted targets and pathways in the alleviation of fluorosis by proanthocyanidins.

## 5. Conclusions

In conclusion, this study is the first to explore the protective effects of GSPE against sodium fluoride-induced liver injury. By integrating network pharmacology predictions with in vivo experiments, we found that the potential molecular mechanisms underlying GSPE’s alleviation of fluoride-induced liver injury are likely related to the mTOR signaling pathway and cellular apoptosis. Specifically, Western blot analysis confirmed our prediction: GSPE mitigates sodium fluoride-induced liver injury by regulating the mTOR/p70s6k signaling pathway, autophagy, and apoptosis levels. These findings suggest that GSPE demonstrates significant potential for preventing and treating sodium fluoride-induced liver injury. Thus, it merits further in-depth investigation as a potential functional compound. This study contributes to our understanding of how natural plant extracts can be used in the prevention and treatment of endemic diseases, thereby advancing public health.

## Figures and Tables

**Table 1:** The binding energy and cavity volume of PC in docking with different receptor molecules.

Protein Receptor	Vina Score (kcal/mol)	Cavity Volume (Å^3^)
TP53	−7.0	78
IL1B	−8.2	185
CASP3	−8.7	1412
IL6	−9.0	134
ALB	−9.5	1986
BCL2	−7.4	285
PARP1	−10.9	2969
mTOR	−9.8	7094

**Table 2:** Alterations in Liver Tissue Toxicity Indicators of Offspring of SD Rats in Each Group ($\bar{x}$ ± s; n = 6).

Group	AST (U/L)	ALT (U/L)
Control	36.14 ± 0.08	25.52 ± 0.24
NaF (100 mg/L)	51.25 ± 6.00 *	42.42 ± 5.47 *
GSPE (400 mg/L)	35.26 ± 1.17	27.6 ± 2.47
NaF (100 mg/L) + GSPE (100 mg/kg·bw)	36.98 ± 0.36 ^#^	25.74 ± 1.25 ^#^
NaF (100 mg/L) + GSPE (200 mg/kg·bw)	36.04 ± 0.63 ^#^	24.71 ± 0.97 ^#^
NaF (100 mg/L) + GSPE (400 mg/kg·bw)	34.63 ± 0.78 ^#^	19.13 ± 1.71 ^##^

Compared with the control group, * *p* < 0.05. Compared with the NaF group, ^#^ *p* < 0.05, ^##^ *p* < 0.01.

## Data Availability

All the data for network analysis are sourced from public databases.

## Supplementary Materials

The following supporting information can be downloaded at: https://www.mdpi.com/article/10.3390/genes16091037/s1, Table S1: Animal weight records. Table S2: Proanthocyanidin targets. Table S3: Disease targets.

## Abbreviations

NaF	Sodium fluoride
PC	Proanthocyanidin
GSPE	Grape seed proanthocyanidin extract
AST	Aspartate aminotransferase
ALT	Alanine aminotransferase
GO	Gene ontology
KEGG	Kyoto encyclopedia of genes and genome

## References

[1] Vasisth D., Mehra P., Yadav L., Kumari V., Bhatia U., Garg R. (2024). Fluoride and its Implications on Oral Health: A Review. J. Pharm. Bioallied Sci..

[2] Whelton H., Spencer A., Do L., Rugg-Gunn A. (2019). Fluoride Revolution and Dental Caries: Evolution of Policies for Global Use. J. Dent. Res..

[3] O’mullane D.M., Baez R.J., Jones S., Lennon M.A., Petersen P.E., Rugg-Gunn A.J., Whelton H., Whitford G.M. (2016). Fluoride and Oral Health. Community Dent. Health.

[4] Larsen L.S., Nyvad B., Baelum V. (2023). Salivary fluoride levels after daily brushing with 5000 ppm fluoride toothpaste: A randomised, controlled clinical trial. Eur. J. Oral Sci..

[5] Adelakun S.A., Akintunde O.W., Ogunlade B. (2021). Fluoride-induced testicular degeneration and sperm quality deteriorations: Salutary role of Cyperus esculentus tubers (tiger nut) extract in animal model. Rev. Int. Androl..

[6] Yu Y.-M., Zhou B.-H., Yang Y.-L., Guo C.-X., Zhao J., Wang H.-W. (2022). Estrogen Deficiency Aggravates Fluoride-Induced Liver Damage and Lipid Metabolism Disorder in Rats. Biol. Trace Elem. Res..

[7] Wang Y., Xiao X., Zhan X. (2018). Antagonistic effects of different selenium sources on growth inhibition, oxidative damage, and apoptosis induced by fluorine in broilers. Poult. Sci..

[8] Tian X., Zhang H., Zhao Y., Mehmood K., Wu X., Chang Z., Luo M., Liu X., Ijaz M., Javed M.T. (2018). Transcriptome analysis reveals the molecular mechanism of hepatic metabolism disorder caused by chromium poisoning in chickens. Environ. Sci. Pollut. Res. Int..

[9] Fujioka Y., Noda N.N. (2021). Biomolecular condensates in autophagy regulation. Curr. Opin. Cell Biol..

[10] Xin Y., Jiang F., Yang C., Yan Q., Guo W., Huang Q., Zhang L., Jiang G. (2017). Role of autophagy in regulating the radios ensitivity of tumor cells. J. Cancer Res. Clin. Oncol..

[11] Ma J., Gao S.-S., Yang H.-J., Wang M., Cheng B.-F., Feng Z.-W., Wang L. (2018). Neuroprotective Effects of Proanthocyanidins, Natural Flavonoids Derived From Plants, on Rotenone-Induced Oxidative Stress and Apoptotic Cell Death in Human Neuroblastoma SH-SY5Y Cells. Front. Neurosci..

[12] Niu Q., Mu L., Li S., Xu S., Ma R., Guo S. (2016). Proanthocyanidin Protects Human Embryo Hepatocytes from Fluoride-Induced Oxidative Stress by Regulating Iron Metabolism. Biol. Trace Elem. Res..

[13] Rodríguez-Pérez C., García-Villanova B., Guerra-Hernández E., Verardo V. (2019). Grape Seeds Proanthocyanidins: An Overview of In Vivo Bioactivity in Animal Models. Nutrients.

[14] Li Y., Zhou T., Liu Z., Zhu X., Wu Q., Meng C., Deng Q. (2025). Air pollution and prostate cancer: Unraveling the connection through network toxicology and machine learning. Ecotoxicol. Environ. Saf..

[15] Zhang Y., Yang Y., Sun Y., Wei Z., Wang D., Chen S., Yang F., Wang J., Kang X. (2025). Assessing the toxicological impact of PET-MPs exposure on IVDD: Insights from network toxicology and molecular docking. J. Environ. Manag..

[16] Cao F., Guo C., Guo J. (2025). Deciphering CSU pathogenesis: Network toxicologyand molecular dynamics of DOTP exposure. Ecotoxicol. Environ. Saf..

[17] Huang S. (2023). Efficient analysis of toxicity and mechanisms of environmental pollutants with network toxicology and molecular docking strategy: Acetyl tributyl citrate as an example. Sci. Total Environ..

[18] Lv Y., Zhang T., Cai J., Huang C., Zhan S., Liu J. (2022). Bioinformatics and systems biology approach to identify the pathogenetic link of Long COVID and Myalgic Encephalomyelitis/Chronic Fatigue Syndrome. Front. Immunol..

[19] Gao K., Hua K., Wang S., Chen X., Zhu T. (2025). Exploring the reproductive exposure risks of phthalates and organophosphates in atmospheric particulate matter based on quantitative structure-activity relationships and network toxicology models. J. Hazard. Mater..

[20] Feng J., Qiu S., Zhou S., Tan Y., Bai Y., Cao H., Guo J., Su Z. (2022). mTOR: A Potential New Target in Nonalcoholic Fatty Liver Disease. Int. J. Mol. Sci..

[21] Kim Y.C., Guan K.L. (2015). mTOR: A pharmacologic target for autophagy regulation. J. Clin. Investig..

[22] Chao X., Wang H., Jaeschke H., Ding W. (2018). Role and mechanisms of autophagy in acetaminophen-induced liver injury. Liver Int..

[23] Opydo-Szymaczek J., Pawlaczyk-Kamieńska T., Borysewicz-Lewicka M. (2022). Fluoride Intake and Salivary Fluoride Retention after Using High-Fluoride Toothpaste Followed by Post-Brushing Water Rinsing and Conventional (1400–1450 ppm) Fluoride Toothpastes Used without Rinsing. Int. J. Environ. Res. Public Health.

[24] Srivastava S., Flora S.J.S. (2020). Fluoride in Drinking Water and Skeletal Fluorosis: A Review of the Global Impact. Curr. Environ. Health Rep..

[25] Ren C., Li H.-H., Zhang C.-Y., Song X.-C. (2022). Effects of chronic fluorosis on the brain. Ecotoxicol. Environ. Saf..

[26] Nabavi S.F., Moghaddam A.H., Setzer W.N., Mirzaei M. (2012). Effect of silymarin on sodium fluoride-induced toxicity and oxidative stress in rat cardiac tissues. An. Acad. Bras. Ciências.

[27] Yang S., Song D., Wang R., Liu M., Tan T., Wang Y., Xie Q., Wang L. (2024). Sodium fluoride-induced autophagy of ameloblast-like cells via the p-ULk1/ATG13/LC3B pathway in vitro. Oral Dis..

[28] Voskarides K., Giannopoulou N. (2023). The Role of TP53 in Adaptation and Evolution. Cells.

[29] Yu J., Ling S., Hong J., Zhang L., Zhou W., Yin L., Xu S., Que Q., Wu Y., Zhan Q. (2023). TP53/mTORC1-mediated bidirectional regulation of PD-L1 modulates immune evasion in hepatocellular carcinoma. J. Immunother. Cancer.

[30] Lu J., Wang X., Tang W. (2009). IL-1β-induced increase in NO production and decrease in mitochondrial membrane potential in rat hepatocytes. J. Southeast Univ. (Med. Sci. Ed.).

[31] Bliźniewska-Kowalska K., Gałecki P., Szemraj J., Su K.-P., Chang J.P.-C., Gałecka M. (2023). CASP3 gene expression and the role of caspase 3 in the pathogenesis of depressive disorders. BMC Psychiatry.

[32] Zhao J.-H., Li J., Zhang X.-Y., Shi S., Wang L., Yuan M.-L., Liu Y.-P., Wang Y.-D. (2023). Confusoside from Anneslea fragrans Alleviates Acetaminophen-Induced Liver Injury in HepG2 via PI3K-CASP3 Signaling Pathway. Molecules.

[33] Zheng X., Wang G., Yuan J., Li N., Yan B., Yan J., Sheng Y. (2022). hsa-miR-34a-5p Ameliorates Hepatic Ischemia/Reperfusion Injury Via Targeting HNF4α. Turk. J. Gastroenterol..

[34] Li D., Qian J., Li J., Wang J., Liu W., Li Q., Wu D. (2022). Dexmedetomidine attenuates acute stress-induced liver injury in rats by regulating the miR-34a-5p/ROS/JNK/p38 signaling pathway. J. Toxicol. Sci..

[35] Chen Y., Zhou X. (2020). Research progress of mTOR inhibitors. Eur. J. Med. Chem..

[36] Zhang J., Zhu Y., Shi Y., Han Y., Liang C., Feng Z., Zheng H., Eng M., Wang J. (2017). Fluoride-Induced Autophagy via the Regulation of Phosphorylation of Mammalian Targets of Rapamycin in Mice Leydig Cells. J. Agric. Food Chem..

[37] Russell R.C., Tian Y., Yuan H., Park H.W., Chang Y.-Y., Kim J., Kim H., Neufeld T.P., Dillin A., Guan K.-L. (2013). ULK1 induces autophagy by phosphorylating Beclin-1 and activating VPS34 lipid kinase. Nat. Cell Biol..

[38] Di R., Yang Z., Xu P., Xu Y. (2019). Silencing PDK1 limits hypoxia-induced pulmonary arterial hypertension in mice via the Akt/p70S6K signaling pathway. Exp. Ther. Med..

[39] Sangaunchom P., Dharmasaroja P. (2020). Caffeine Potentiates Ethanol-Induced Neurotoxicity Through mTOR/p70S6K/4E-BP1 Inhibition in SH-SY5Y Cells. Int. J. Toxicol..

[40] Alemasova E.E., Lavrik O.I. (2019). Poly(ADP-ribosyl)ation by PARP1: Reaction mechanism and regulatory proteins. Nucleic Acids Res..

[41] Xi H., Wang S., Wang B., Hong X., Liu X., Li M., Shen R., Dong Q. (2022). The role of interaction between autophagy and apoptosis in tumorigenesis (Review). Oncol. Rep..

[42] Li Y., Liu Y., Yi J., Li Y., Yang B., Shang P., Mehmood K., Bilal R.M., Zhang H., Chang Y.-F. (2021). The potential risks of chronic fluoride exposure on nephrotoxic via altering glucolipid metabolism and activating autophagy and apoptosis in ducks. Toxicology.

[43] Angwa L.M., Nyadanu S.D., Kanyugo A.M., Adampah T., Pereira G. (2023). Fluoride-induced apoptosis in non-skeletal tissues of experimental animals: A systematic review and meta-analysis. Heliyon.

